# An Investigation of Emotion Recognition and Theory of Mind in People with Chronic Heart Failure

**DOI:** 10.1371/journal.pone.0141607

**Published:** 2015-11-03

**Authors:** Tina Habota, Skye N. McLennan, Jan Cameron, Chantal F. Ski, David R. Thompson, Peter G. Rendell

**Affiliations:** 1 Cognition and Emotion Research Centre, Australian Catholic University, Melbourne, Australia; 2 Centre for the Heart and Mind, Australian Catholic University, Melbourne, Australia; University of Tuebingen Medical School, GERMANY

## Abstract

**Objectives:**

Cognitive deficits are common in patients with chronic heart failure (CHF), but no study has investigated whether these deficits extend to social cognition. The present study provided the first empirical assessment of emotion recognition and theory of mind (ToM) in patients with CHF. In addition, it assessed whether each of these social cognitive constructs was associated with more general cognitive impairment.

**Methods:**

A group comparison design was used, with 31 CHF patients compared to 38 demographically matched controls. The Ekman Faces test was used to assess emotion recognition, and the Mind in the Eyes test to measure ToM. Measures assessing global cognition, executive functions, and verbal memory were also administered.

**Results:**

There were no differences between groups on emotion recognition or ToM. The CHF group’s performance was poorer on some executive measures, but memory was relatively preserved. In the CHF group, both emotion recognition performance and ToM ability correlated moderately with global cognition (*r* = .38, *p* = .034; *r* = .49, *p* = .005, respectively), but not with executive function or verbal memory.

**Conclusion:**

CHF patients with lower cognitive ability were more likely to have difficulty recognizing emotions and inferring the mental states of others. Clinical implications of these findings are discussed.

## Introduction

Chronic heart failure (CHF) is a complex condition characterized by an underlying structural abnormality that impairs the function of the heart to deliver sufficient blood flow to meet the metabolic needs of the body and brain [1]. In many patients with CHF, accumulated ischemic damage to the brain results in secondary cognitive impairment [2,3]. The level of cognitive impairment varies from patient to patient, but some degree of dysfunction is seen in up to 80% of patients in select CHF populations [4,5]. Over the past few decades, multiple neuropsychological studies have established that people with CHF are affected by deficits in cognitive processes such as executive function, memory, and attention (for review see, [5]). However, no study has assessed social cognition in this group.

Core aspects of social cognition are emotion recognition, which is the ability to perceive and correctly distinguish emotions displayed by others [6], and Theory of Mind (ToM), which is the ability to make inferences about the mental states of others [7]. These two processes of social cognition are vital because they facilitate effective social interaction and allow people to form and maintain strong relationships with others [8] by enabling them to understand subtle social cues [9]. Profound examples of deficits in these processes are seen in people with autism spectrum disorders [10] and schizophrenia [11,12].

In clinical groups, deficits in social cognition have been linked to poor functional outcomes, for example poor community and/or psychological functioning [13,14]. Therefore, social cognition may be particularly important for patients with CHF who experience debilitating physical symptoms that impact on their physical and emotional wellbeing, thereby increasing their need for support from others [15,16]. Social cognitive impairment may also contribute to isolation through poor social functioning [13]. This is important because social isolation is a significant predictor of mortality in CHF, while social support increases overall quality of life [17].

The overarching aim of this study was to examine emotion recognition and ToM in patients with CHF. It is possible that the deficits CHF patients experience with other cognitive abilities also extend to deficits in social cognition. This is because the neuropathology observed in these patients involves white matter hyperintensities and reduced grey matter [2,18] in regions of the brain that are implicated in emotion recognition and ToM, including the prefrontal cortex and the limbic system (temporal lobe) [19,20]. In particular, the observed white matter pathology is associated with disconnection within fronto-subcortical brain tracts [21] known to be involved in the processing of emotional signals [22,23].

The second aim of the study was to assess whether deficits in emotion recognition and ToM were associated with more general cognitive impairment. Although general cognition and social cognition are supported by different neural circuits [24–26], the process of understanding others’ thoughts and emotions has been shown to make substantial demands on cognitive control processes, such as inhibition and mental flexibility [27–29]. Given that executive control mechanisms are supported by frontal brain regions [26], which are amongst the most affected in CHF, it might be that in the context of CHF, any observed deficits in emotion recognition and ToM are related to more general cognitive difficulties. Indeed, a relationship between emotion recognition and/or ToM and general cognition has been observed in other clinical populations [30,31].

To address each of these aims, a group of CHF patients was compared to a group of matched controls. It was hypothesized that the CHF group would show deficits in emotion recognition and ToM compared to the group of healthy controls. It was also predicted that within the CHF group, emotion recognition and ToM would positively correlate with global cognition, executive function, and verbal memory.

## Methods

This research was approved by the Human Research Ethics Committees at Eastern Health and the Australian Catholic University. All participants provided written informed consent.

### Participants

The CHF group was recruited from a pool of participants taking part in a larger study (*n* = 72); 13 participants were paid AUD $10 per hour, and the rest were volunteers. To be eligible for the parent study participants had to be aged over 18, and actively engaged in a nurse-led CHF management program at one of three public hospitals in metropolitan Melbourne, Australia. All recruited participants had a confirmed diagnosis of CHF. Specifically, cardinal symptoms and clinical features of congestion, and objective evidence of cardiac impairment on echocardiogram [1].

Participants with CHF were excluded if they resided in a high care residential aged facility, had a terminal diagnosis, a documented history of dementia, or could not read English. All 72 participants were approached from the parent study; 25 declined, and five were unreachable. No participants had head injury or psychiatric illness. We screened participants’ global cognition; initially, we recruited 42 participants but excluded six who could not complete the primary measures because they either declined or ran out of time. Another five participants were excluded who showed signs of potential dementia as operationalized by a score lower than 82 on the Addenbrooke’s Cognitive Examination—Revised (ACE-R) [32]. The final sample of CHF patients included 31 adults. A subset of this final sample has been reported on previously [33], but not with regards to social cognitive performance.

The control group was recruited from the general community; 13 participants were paid AUD $10 per hour, the rest were volunteers. Participants in the control group were excluded if they reported a history of CHF or neurological disease, had recent treatment (past three months) for an acute cardiovascular problem, or could not read English. We initially recruited 43 participants, but excluded four who could not complete the primary measures, and one who had an ACE-R score below the cut off. The final control group included 38 adults.

### Materials

#### Health

The *New York Heart Association* (NYHA) classification [34] was used as an index of functional status. The NYHA is an extensively used and validated clinical measure. Classification I indicates no limitations and classification IV indicates the worst functional status.

The *Charlson Co-morbidity Index* [35] was used to assess the severity of co-morbid conditions. Overall index scores are categorized as mild, moderate, or severe; higher scores indicate more severe co-morbidity.

#### Global cognition

The *Addenbrooke’s Cognitive Examination—Revised* [32] is a test of global cognition. The ACE-R is a reliable and sensitive test of early cognitive dysfunction [36] and was used to identify and exclude participants with possible dementia (scores <82). Higher scores indicate better cognitive functioning.

#### General intelligence

The *National Adult Reading Test* (NART) [37] was used as an index of premorbid intelligence. Standardized IQ scores were calculated using the formula in the administration manual [37].

#### Psychopathology

The *Hospital Anxiety and Depression Scale* [38] was used to screen for symptoms of anxiety and depression. The level of emotional symptomology was assessed separately for anxiety and depression (seven items each). Higher scores indicate higher levels of symptomology.

#### Executive function—cognitive flexibility

The *Trail Making Test* (TMT) [39] was used to assess cognitive flexibility. The Trails B minus Trails A difference score was used as an index of cognitive flexibility [40]. Lower scores indicate better performance.

#### Executive function—inhibition

The *Hayling Sentence Completion* test [41] was used to assess cognitive inhibition. A total score was obtained by tallying errors and time taken (in seconds) to complete the task. Standardized scores were calculated; higher scores indicate better performance.

#### Executive function—initiation

The final measure of executive functioning was *verbal fluency*, which was extracted from the ACE-R and used to assess cognitive initiation. Two types of verbal fluency were assessed; phonemic and categorical. In the phonemic task, participants were given one minute to orally generate as many words beginning with the letter ‘P’ as they could, excluding proper nouns and the same word with a different suffix. In the categorical task, participants were given one minute to name as many animals as they could. A composite verbal fluency score was used in the present study. Higher scores indicate better performance.

#### Verbal memory

The *Rey Auditory Verbal Learning Test* (RAVLT) [42] was used to measure verbal memory. A single composite score for the first five trials was used as the measure of immediate recall. The total number of correctly recalled words after a 20-minute delay was used as a measure of delayed recall. Higher scores indicate better performance.

### Primary Measures

#### Emotion recognition

The *Ekman 60 Faces* test [43] was used to assess recognition of six basic human emotions; happy, anger, sadness, disgust, surprise, and fear. Participants were presented with 60 slides, featuring extensively and universally validated photographs of human faces [44]. Participants were asked to choose one of six emotions that best described the emotion that the person in the picture displayed. This measure takes approximately 15 minutes to administer, and it has been used extensively to assess emotion recognition in various groups.

#### Theory of Mind

The *Reading the Mind in the Eyes* test [45] was used to assess ToM. Participants were presented with 36 black and white images of the eye region of human faces, and asked to indicate which of four given emotional states each image best represents. In comparison to measures of emotion recognition, which require participants to identify basic facial expressions, this task required participants to identify more complex and finely nuanced mental states (e.g., “perplexed”, “flirtatious”). This measure is administered in approximately 10 minutes. It is a reliable and valid measure of social cognitive dysfunction, and it is commonly used to assess ToM in various clinical and non-clinical groups [46].

### Procedure

Participants with CHF were tested approximately three months after recruitment into the parent study. The delay of three months was built in to ensure that participants were medically stable when they completed the neuropsychological assessment. Participants were tested in a quiet room, either at their residences, in a hospital consultation room, or in a university-testing lab in a single session, lasting approximately two hours.

### Design and data analysis

This study used a matched-group comparison design. Missing value analysis was conducted, which showed that data was missing at random. Therefore missing data were not substituted. Descriptive statistics were generated for all variables. Univariate analyses were conducted to assess group differences on the background cognitive measures, and the ToM task. A mixed two-way ANOVA was used to examine differences in performance on the emotion recognition measure.

Pearson correlations were used to examine associations between the social cognitive measures and each of the other cognitive measures. Before undertaking the correlational analyses, all variables were assessed for normality. In the CHF group, all variables were normally distributed. In the control group, the Ekman Faces score was negatively skewed. Skewness was corrected by adjusting one outlier (which was 3 standard deviations below the mean) to two standard deviations below the mean [47].

To reduce the possibility of Type I error, we created a composite executive function score given that the three measures of executive function (TMT, Hayling, verbal fluency) were correlated: TMT with Hayling (*r* = -.38, *p* = .003), TMT with verbal fluency (*r* = -.25, *p* = .048), and Hayling with verbal fluency (*r* = .41, *p* = .001). The composite executive function score was created by converting scores on the three measures of executive function to *z* scores, reversing-coding the TMT such that higher scores indicated higher performance, then calculating a mean *z* of the three scores for each participant. The two measures of verbal memory (immediate and delayed recall, RAVLT) were also correlated (*r* = .79, *p* < .001) so a composite verbal memory score was created using the same approach. Both composite scores were normally distributed.

## Results

### CHF group characteristics

The CHF group consisted of adults aged 40 to 86 (*M* = 69.77, *SD* = 11.23) who were predominantly male (65%). Table 1 shows that the majority of the CHF sample was classified as functional classification II on the NYHA. Systolic and ischemic CHF were the most common etiologies in this group, and hypertension was the most frequently reported risk factor. On average, participants had a moderate level of comorbid disease burden (Charlson Comorbidity Index mean = 3.48, *SD* = 2.03), and the average length of time living with CHF was three years (*M* = 36.17 months, *SD* = 55.49).

**Table 1:** Clinical Characteristics of the CHF Group.

*Health characteristics*	*n*	*%*
*NYHA classification*		
Class I	2	6.5
Class II	18	58.1
Class III	10	32.3
Class IV	1	3.2
*Heart failure type*		
Systolic	22	71.0
Diastolic	4	12.9
Mixed	3	9.7
Unspecified	2	6.5
*Heart failure etiology*		
Ischemic	17	54.8
Non ischemic	8	25.8
Idiopathic	1	3.2
Other	5	16.1

*Note*. NYHA = New York Heart Association.

### Group comparisons on demographics and cognition

Table 2 shows that there was a trend towards higher proportions of cardiac risk factors in the CHF group, but these group differences were not significant (all *p*s > .060). Table 2 also shows that the two groups were closely matched in gender distribution, age, education, and estimated IQ as indexed by the NART. Independent samples *t*-tests were conducted to examine differences between groups on cognitive measures (Table 2). The control group performed significantly better on two of the three measures of executive function; cognitive flexibility *t*(62) = 3.07, *p* = .003, and cognitive inhibition *t*(61) = 5.88, *p* < .001, but not on either measures of verbal memory. The control group reported more symptoms of anxiety *t*(67) = 2.23, *p* = .029.

**Table 2:** Participant Characteristics.

	*CHF group*			*Control group*				
	*n*	*%*		*n*	*%*		*χ* ^*2*^	
Proportion of men (*%*)	20	65.0		27	71.0		0.34	
*Cardiac risk factors* (*%*)								
Hypercholesterolemia	13	41.9%		15	39.5%		0.04	
Hypertension	21	67.7%		18	47.4%		2.88	
Smoking	13	41.9%		8	21.1%		3.52	
Diabetes	7	22.6%		4	10.5%		1.85	
Obesity	5	16.1%		2	5.3%		2.21	
*Demographic* (*M*)		*M*	*SD*		*M*	*SD*	*t*	*d*
Age (years)	31	69.77	11.23	38	67.13	7.53	1.12	0.28
Education (years)	31	11.65	3.74	38	13.07	3.57	1.61	0.39
Estimated IQ	30	112.54	5.89	38	114.33	6.38	1.19	0.29
*Global cognition and mental health*								
Global cognition (ACE-R)	31	91.00	4.89	38	92.08	4.55	0.95	0.23
Anxiety (HADS)	31	6.03	3.73	38	8.29	4.51	2.23*	0.55
Depression (HADS)	31	5.19	2.91	38	5.55	3.29	0.48	0.12
*Executive functions*								
Cognitive flexibility (TMT)	27	74.83	35.17	37	49.19	31.35	3.07**	0.77
Inhibition (Hayling)	28	2.89	2.01	35	5.57	1.61	5.88***	1.47
Initiation (Verbal fluency)	31	29.94	6.51	38	32.21	7.85	1.29	0.31
*Verbal memory* (RAVLT)								
Immediate recall	26	41.58	8.89	38	45.00	8.96	1.51	0.38
Delayed recall	24	8.83	2.51	38	9.13	2.84	0.42	0.11

*d* = Cohen’s *d* index of effect size. Effect sizes: small = 0.2; medium = 0.5; large = 0.8 [48].

* *p* < .05.

** *p* < .01.

*** *p* < .001.

*Notes*. ACE-R = Addenbrooke’s Cognitive Examination—Revised; HADS = Hospital Anxiety Depression Scale; RAVLT = Rey Auditory Verbal Learning Test; TMT = Trail Making Test (B minus A).

### Group comparisons on measures of emotion recognition and ToM

#### Emotion recognition: Ekman Faces test

**Fig 1:**
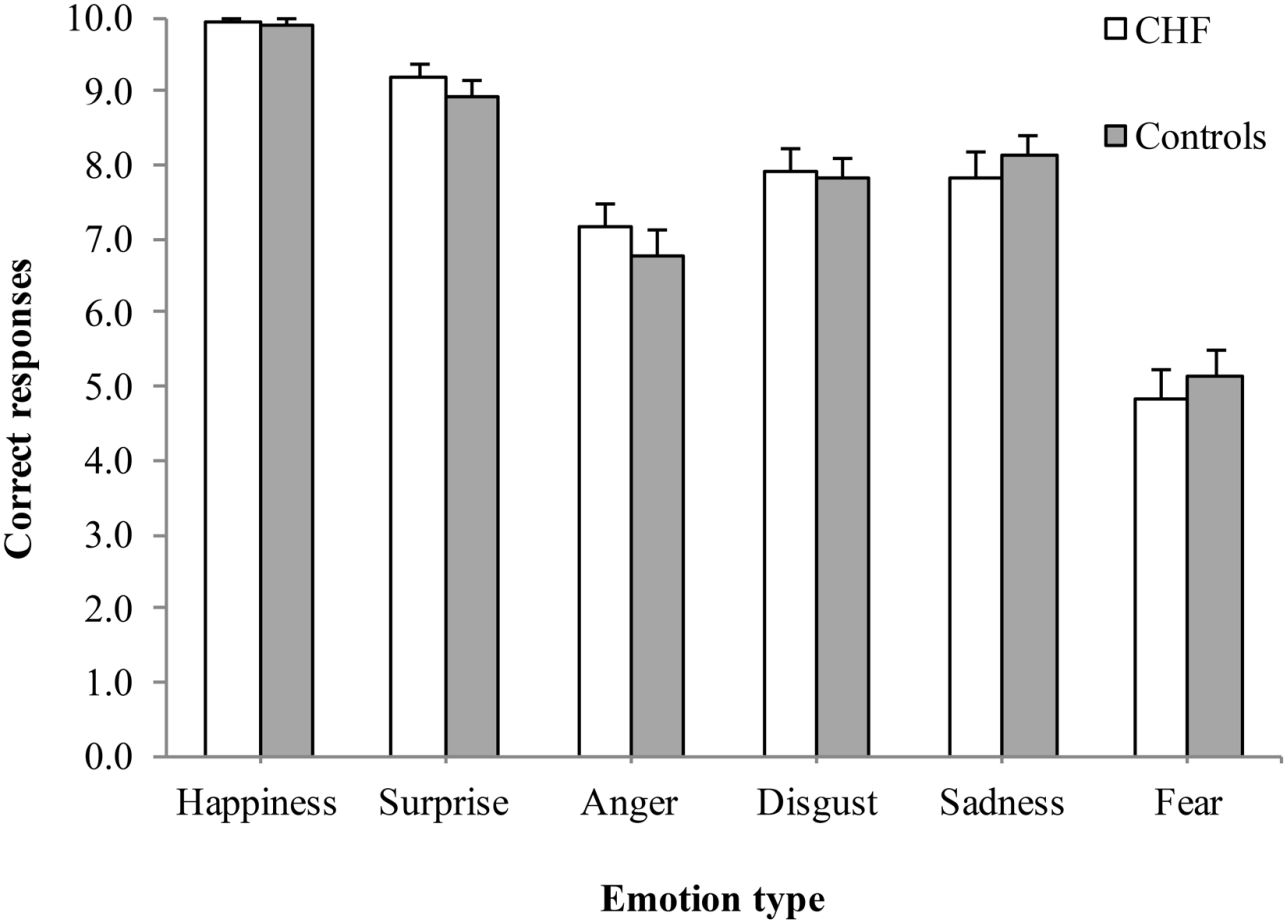
Mean number of correct responses for each emotion type on the Ekman Faces test for the CHF and control groups.

Fig 1 shows the results of the Ekman Faces test as a function of *group* (CHF, control) and *emotion type* (happiness, surprise, anger, disgust, sadness, fear). These data were analyzed with a mixed 2 x 6 ANOVA with the between-groups variable of *group* and the within-groups variable of *emotion type*. Mauchly’s test indicated that the sphericity assumption was violated; therefore the Huynh-Feldt correction was used. Of primary interest, there was no significant main effect of group *F*(1, 67) = 0.01, *p* = .932 ηp^2^ < .001, and no interaction effect *F*(4.38, 293.15) = 0.76, *p* = .566, η_p_^2^ = .011, which indicates that recognition of basic emotions did not differ as a function of group status. Of secondary interest was the main effect of emotion type, *F*(4.38, 293.15) = 99.99, *p* < .001, η_p_^2^ = 0.60. Post hoc revealed that for all participants, the recognition accuracy significantly differed for each comparison of each type of emotion, with the order from best to worst recognized being: happiness (*M* = 9.91, *SD* = 0.33), surprise (*M* = 9.04; *SD* = 1.14), sadness (*M* = 7.99; *SD* = 1.82), disgust (*M* = 7.87; *SD* = 1.62), anger (*M* = 6.94, *SD* = 1.91), and fear (*M* = 4.99, *SD* = 2.17). The one exception was that recognition accuracy did not differ for the comparison of sadness and disgust.

#### Theory of Mind: Mind in the Eyes test

An independent samples *t*-test revealed that the groups did not significantly differ on their ability to accurately infer the mental states of others, *t*(66) = 0.63, *p* = .450, *d* = 0.18 (CHF *M* = 23.87, *SD* = 4.39; controls *M* = 23.08, *SD* = 4.23). The effect size was less than the cut off for a small Cohen’s *d* (0.2) [48].

### Correlations between emotion recognition and ToM and other cognitive measures

Pearson correlations were computed separately for the CHF group and controls to assess the relationship between emotion recognition and ToM and the other cognitive measures. Separate correlations were run for the Ekman Faces test and the Mind in the Eyes test (Table 3). All correlations were in the expected direction for both groups with better cognitive performance associated with better social cognition performance. In the CHF group, both emotion recognition and ToM showed significant moderate positive correlations with global cognition (*r* = .38 *p* = .034; *r* = .49 *p* = .005, respectively). However, neither emotion recognition nor ToM significantly correlated with executive function or verbal memory. In the control group, neither executive function nor verbal memory significantly correlated with the Ekman Faces test and the Mind in the Eyes test, however there was a significant correlation between the Ekman Faces test and the Mind in the Eyes test (*r* = .45, *p* = .004), as might be expected.

**Table 3:** Relationships between Emotion Recognition (Ekman) and ToM (Mind in the Eyes) Scores and Cognitive Measures.

	Ekman Faces				Mind in the Eyes			
	CHF		Controls		CHF		Controls	
	*n*	*r* (*p*)	*n*	*r* (*p*)	*n*	*r* (*p*)	*n*	*r* (*p*)
Global cognition (ACE-R)	31	.38 (.034) *	38	.10 (.565)	31	.49 (.005) **	38	.07 (.659)
Executive function	26	.02 (.957)	34	.15 (.410)	26	.14 (.495)	34	.25 (.158)
Verbal memory	24	.30 (.154)	38	.21 (.208)	24	.21 (.327)	38	.11 (.530)
Mind in the Eyes	31	.29 (.104)	38	.45 (.004) **		-		-

* *p* < .05.

** *p* < .01.

*Note*. ACE-R = Addenbrooke’s Cognitive Examination—Revised; Executive function and verbal memory are both composite scores.

## Discussion

A large body of research has shown that people with CHF present with deficits in a range of cognitive abilities [3,49]. In the present study the CHF group’s cognitive performance varied across cognitive domains. People with CHF showed deficits in some, but not all, cognitive functions, with relatively preserved memory function. No previous studies have investigated whether these deficits might extend to social cognition. The present study compared the emotion recognition and ToM abilities of people with CHF to a group of demographically matched controls. Contrary to expectations, the findings indicated that the performance of the two groups did not differ on either aspect of social cognition. This is also the first study to examine the association between social cognition and more general cognition. Importantly, in the CHF group, people with lower global cognitive ability were more likely to have difficulty recognizing emotions and inferring the mental states of others, as expected. However, contradictory to our prediction, emotion recognition and ToM were not significantly correlated with measures of executive function or verbal memory.

The absence of group differences in emotion recognition and ToM is surprising because people with CHF are affected by diffuse damage to neural structures, including frontal and temporal regions [2,18], which have specifically been implicated in both of these social cognitive processes [19,20]. The lack of group differences is further surprising because similar diffuse neural damage and widespread cognitive impairment is seen in other neurocognitive disorders, including people with traumatic brain injury [31,50,51], autism spectrum disorders [10,52] and multiple sclerosis [30,53,54]. Each of these groups has shown significant deficits in general cognition, but also in emotion recognition and ToM. Furthermore, social cognition deficits have also been observed in a range of neuropsychiatric disorders, most commonly schizophrenia [11,12], but also mood disorders, such as major depression and anxiety for review see, [55]. Thus, the common finding that CHF patients are affected by elevated rates of depression and anxiety [56], might have been expected to further increase their vulnerability to social cognition deficits.

There are several likely explanations for the null findings of this study. In the CHF group, correlations between emotion recognition and ToM with variables that had missing data (i.e., composite verbal memory *n* = 24; executive function *n* = 26) were underpowered; a post-hoc power analysis showed that the study power was .42 for a medium effect size (*r* = .30; the strongest correlation observed with a reduced sample size). Additionally, participants were a select and relatively high functioning group, cognitively and symptomatically. Specifically, we were interested in the performance of non-demented participants and therefore excluded anyone who showed signs of dementia. In addition, 64.6% of the CHF sample had no, or only mild, heart failure symptoms, and the overall subjective rating of depression was within the normal range. Thus, in the wider CHF population, where medical [2,57–59] and emotional [60–62] symptoms are often more severe, brain pathology may also be more severe. Consequently, the ability to successfully recognize emotions and make inferences about the mental states of others is likely to be more impaired in CHF patients with greater comorbidity and worse functioning.

Finally, we chose measures of emotion recognition and ToM that have been used extensively with other clinical groups. However, they may not have been sensitive enough to detect subtle group differences because the CHF group was high functioning. Other studies have found that traditional and static measures of emotion recognition and ToM, like those used in the present study, do not always detect deficits that are picked up by dynamic measures [63–65]. Thus, future research could extend this study by using dynamic and/or morphed images (emotionally neutral expressions that are morphed into expressions of a specific emotion; e.g., [66]) and include measures of reaction time. These measurement approaches could be more sensitive to subtle deficits in emotion recognition in high functioning groups.

Although preliminary, the significant correlations between global cognition and emotion recognition and ToM suggest that CHF patients with lower cognitive functioning are also more likely to have social cognitive deficits. If these findings are confirmed by other studies, then patients with poor cognitive function might require tailored intervention that focuses on improving psychosocial functioning. This is an important consideration because a key factor in successful CHF self-care is social support [67]. The available evidence has shown that social isolation and lack of social support are associated with increased risk of rehospitalization and death [68–70]. Therefore, for patients with low global cognition, emotion recognition and ToM may indirectly impact self-care decision-making and quality of life through impoverished relationships or social isolation. Indeed, this indirect effect has been reported in people with schizophrenia where general cognitive abilities affected social abilities, which consequently exerted a negative influence on general functioning and quality of life [13,14].

In conclusion, in this group of high functioning CHF patients, which was matched to the control group on many important characteristics, capacity for emotion recognition and ToM was not found to be impaired, but each social cognitive construct was related to global cognition. Considering these preliminary findings, it seems likely that people with CHF who present with low general cognitive ability may also be affected by difficulties with accurately recognizing emotions and inferring the mental states of others. This information is important given that psychosocial status, including social support and isolation are important influences of CHF self-care [67].
